# Progress report on the single transducer ultrasonic thermometer using electrostatic actuator

**DOI:** 10.1016/j.heliyon.2024.e26418

**Published:** 2024-02-19

**Authors:** Michal Voldán, Libor Husník, David Mahovský

**Affiliations:** a Czech Metrology Institute, Regional Branch Praha, Department of Primary Metrology of Thermal Quantities, Radiová 1136/3, 102 00 Praha 10, Czech Republic; b Czech Technical University in Prague, Faculty of Electrical Engineering, Technická 2, 166 27 Praha 6, Czech Republic

**Keywords:** Practical acoustic thermometry, Temperature measurement, Ultrasonic thermometer

## Abstract

This article presents improvements to the practical acoustic thermometer of the Czech Metrology Institute that enable measurements with argon gas. Through a careful comparison with standard platinum resistance thermometers, the device shows its potential as a reference instrument for the calibration of industrial thermometers with traceability to the thermodynamic temperature scale. The extended temperature range, now from −80 to 80 °C, is characterized by the measurement uncertainty of well below 0.15 °C. Challenges arise above 100 °C, where the microphone approaches its maximum permissible temperature, which leads to discrepancies in the time of flight (*τ*) measurement results. The lower temperature limit is constrained by temperature-controlled enclosures, suggesting possible redesigns for future improvements. Modifications to the waveguide cover could enable isothermal measurements at higher pressures, which would allow calculation of the speed of sound (*u*_0_). In addition, the upper temperature limit could be extended by lengthening the waveguide or installing a special high-temperature microphone. These results underline the versatility of PAT and provide insights for further improving its effectiveness over a wider temperature range.

## 1 Introduction

Since the temperature *T*_90_ (given by the ITS-90, see Refs. [1,2]) is formed by phase transitions of several Fixed Points (detail in Refs. [3,4]) and interpolation procedures, conventional thermometers carry out an indirect *T*_90_ measurements.

The Boltzmann constant *k*_B_, which has a fixed value, serves as the basis for the definition of the thermodynamic temperature *T* [5]. Well-characterised and correctly described physical systems that allow the direct establishment of state equations without the need to include unknown temperature-dependent parameters are called primary thermometers [6]. Such systems are capable of measuring *T* directly and independently on any temperature range or artefact with an accuracy high enough to represent the realisation of the Kelvin unit or to determine errors in the ITS-90 mathematical apparatus [6].

One possible embodiment of a primary thermometer is the acoustic gas thermometer (AGT) which determines *T* based on the speed of sound [7]. The speed of sound is calculated from measurements of the acoustic resonance frequencies (details in Ref. [8]) of a cavity with a simple geometry filled with a monoatomic low-density gas (see Ref. [9]). The cavities used in this technique are usually made of stainless steel or copper [10] to provide rigid walls. As mentioned in Ref. [11], the relationship between the temperature and zero-pressure sound speed *u*_0_ is

(1) $$u_{0}^{2}\left(T\right)=\frac{\gamma RT}{M}\text{,}$$

where *R* is the molar gas constant, *M* the average molar mass and γ the ratio of heat capacities *c*_*p*_/*c*_*V*_. To extrapolate to the theoretical *u*_0_ value, a series of measurements at different pressures is usually performed (see Ref. [8]).

Primary thermometers are currently not designed for the calibrations of conventional thermometers [6] and therefore several examples of practical acoustic thermometers (PAT) have already been presented, e.g. in Refs. [[12], [13], [14], [15]]. Even if the measurement uncertainty does not reach the sub-milikelvin values, such a device can ensure traceability to the thermodynamic temperature scale as a reference thermometer for calibrations by comparison.

PAT setups use a waveguide for a process known as the "pulse-echo method". This involves measurement of the “time of flight” *τ* of short acoustic pulses to determine the speed of sound. By substituting the average air parameters, Eq. (1) can be simplified as

(2) $$u_{air}=331.41+0.61∙t\text{,}$$

with *t* = *T*-273.15 in °C. The simplest method for determining *τ* from measured signals is "threshold crossing” [16], but it is the least accurate technique. Usually the phase detection method (see Refs. [17,18] or [19]) or the correlation method [20] are used.

The latest advances in the field of practical acoustic thermometry are published in Ref. [21]. The device uses a spiral closed waveguide with two cross-sections and a ½" pre-polarized free-field microphone to measure the speed of sound *u* at pressures up to 400 kPa in argon gas in the range of (0–70) °C with an uncertainty of up to 65 mK.

In this paper, the setup originally presented in Ref. [22] by the Czech Metrology Institute (CMI) and the Czech Technical University in Prague is modified and characterised for the temperature range (−80 to 80) °C using argon gas.

## 2 practical part

**Fig. 1:**
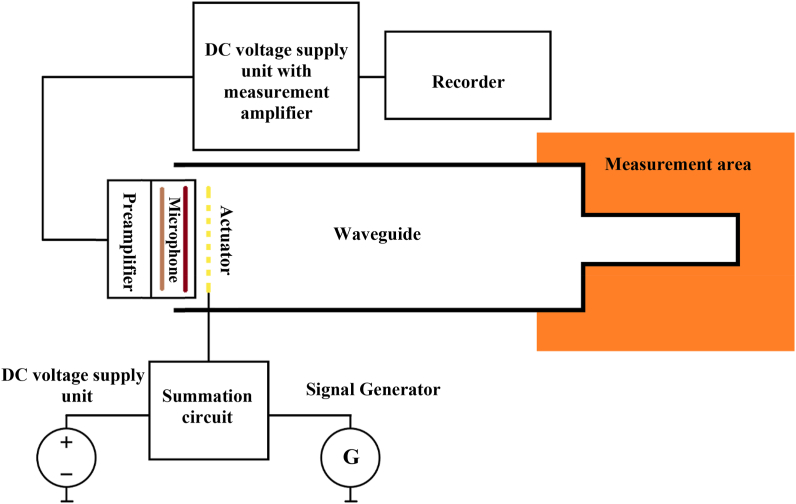
Principal scheme of the practical acoustic thermometer (PAT) at CMI.

The functional principle of the PAT is shown in Fig. 1. The stainless-steel waveguide filled with gas consists of two parts with different cross-sections. One cross-section with a smaller diameter is completely immersed into the environment whose temperature is to be measured. On the opposite side of the waveguide there is the electrostatic microphone with an acoustically transparent actuator, which is positioned a short distance from the diaphragm. This arrangement is well known and is often used in the electrostatic calibration method for calibrating electrostatic microphones, see e.g. Ref. [23]. The electrostatic microphone consists of a variable capacitance composed of a counter electrode and a diaphragm. The polarizing voltage applied between them generates the electrostatic field. When the diaphragm with the surface *S* moves due to the acoustic pressure acting on it, the capacitance of the set changes and if the charge *Q* is kept constant, the displacement of the diaphragm *Δd* is proportional to the changes in the voltage *Δu* which is expressed by Eq. (3) as a signal proportional to the acoustic pressure and symbol ε denotes the permittivity of the medium between the electrodes.

(3) $$\Delta u=\frac{Q}{\varepsilon S}\Delta d$$

in the electrostatic calibration method, another electrode is placed in front of the diaphragm, creating another electrostatic transducer. This transducer drives the diaphragm with the help of the electrostatic force *F* which can be easily determined from its parameters as

(4) $$F=\frac{\varepsilon {SU}_{0}}{d^{2}}u\text{,}$$

where *d* is the distance between the electrode and the diaphragm, *U*_0_ is the polarization voltage and *u* being the signal voltage applied to the driving electrode. The calibration can then be performed. The same arrangement can also be used as an actuator to transmit the measuring sound signal towards the closed end of the waveguide. The same diaphragm, driven by the force described by Eq. (4) transmits the sound and also detects the acoustic response, which consists of the reflections of the emitted sound from two distances (the change in diameter and the end of the waveguide). The value of *τ* is then calculated from the difference in the arrival times of the respective signals, representing the two reflections.

The electrostatic microphone with the driving electrode used as an actuator in the so called “single-acting arrangement” (there is only one back electrode driving the diaphragm) shows the signal distortion by the second harmonic, since the electrostatic force depends on the second power of the distance between the diaphragm and the electrode. However, this does not affect our method as it only uses the time of flight of signals whether or not distorted. In double acting arrangements, which is usual in electrostatic drivers, the distortion by second harmonic is cancelled.

The LabVIEW application manages the entire process of the PAT operation.

The first comparison of CMI PAT with the reference standard platinum resistance thermometer (SPRT) was performed (in Ref. [22]) in air and at ambient air pressure using Eq. (2) to calculate theoretical *τ* values from SPRT thermometer results.

**Fig. 2:**
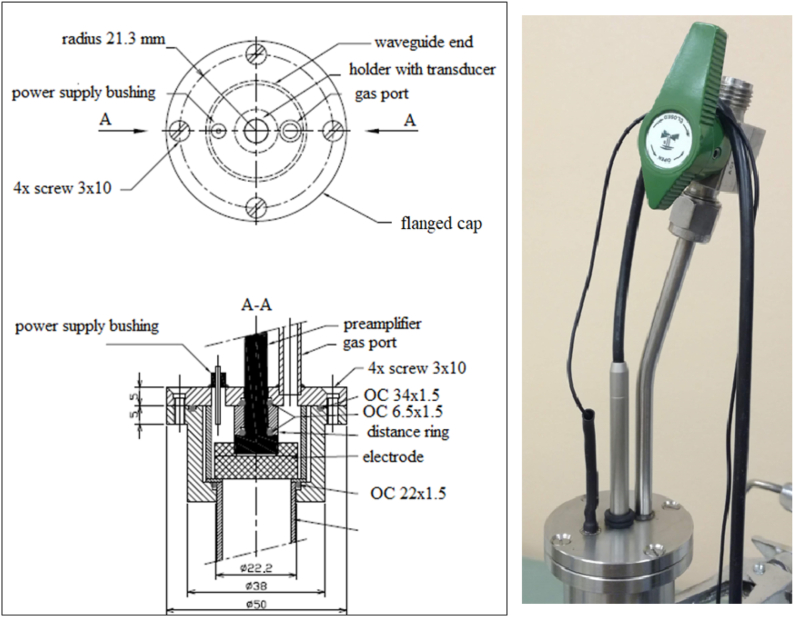
The lid design (left) and physical form (right).

However, Eq. (1) must be used to measure *T* and this applies to monoatomic gases (such as helium, neon, argon, krypton, xenon and radon) with properties similar to those of an ideal gas. The choice of argon as the cheapest representative is normally sufficient for PAT. To enable its use, a stainless-steel cover (design in Fig. 2) was added to the waveguide. It also mechanically stabilises the microphone, the preamplifier and the actuator (perforated metal electrode).

**Fig. 3:**
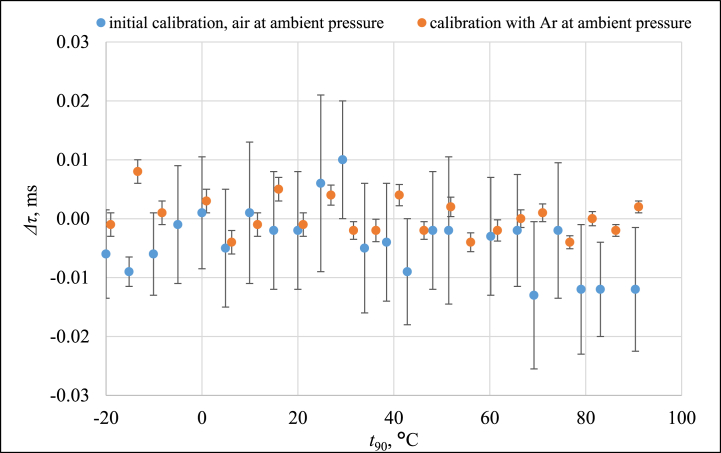
SPRT and PAT comparison results in the range of (−20 to 90) °C and its standard deviation.

The comparison between PAT and SPRT was repeated to investigate the effects of enclosing the waveguide and replacing the air medium with 99.999 % pure argon. The measurement results for ambient pressure and the results from Ref. [22] are shown in Fig. 3. Here, *Δτ* denotes the difference between the measured *τ* and the *τ* calculated from the SPRT temperature measurements. The effect of sealing the waveguide and using argon gas is mainly of minimization of the standard deviation of the measurement results.

**Fig. 4:**
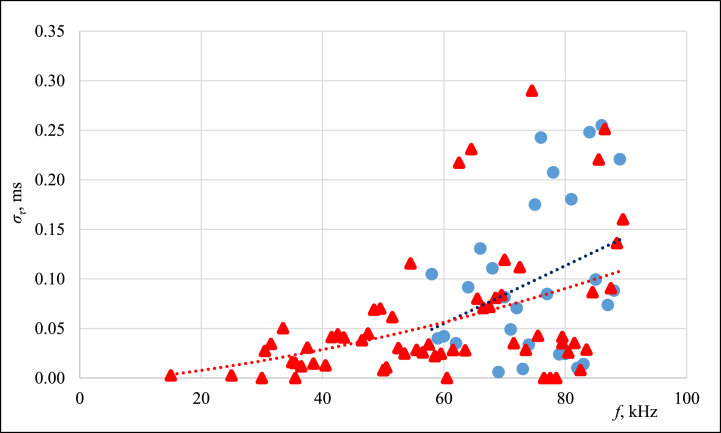
Influence of sound frequency on the standard deviation σ_τ_ of the measured τ in calibration bath at 0 °C (in blue) and in melting point of ice (in red). (For interpretation of the references to colour in this figure legend, the reader is referred to the Web version of this article.)

The frequency of 75 kHz was used in previous work (published in Ref. [22]) for exhibiting the smallest *Δτ* values among the frequencies tested. This time, the influence of frequency on the standard deviation (as shown in Fig. 4) was measured in the range of (15–90) kHz at the melting point of ice (0 °C) and using a calibration ethanol bath at 0 °C.

The data shown in Fig. 4 are supported by the theory e.g., in Ref. [24] (Chapter 5), that at frequencies in the range from 590 Hz to 14.82 kHz at −20 °C and from 710 Hz to 17.75 kHz at 90 °C for the largest radial internal dimension of the waveguide $l_{T}$, the wavelength λ and the biggest longitudinal internal dimension

(5) $$l_{T}<\lambda <l_{l}\text{,}$$

and the plane waves propagate inside the waveguide, since the radial modes are not excited. For all other frequencies

(6) $$\lambda <l_{T}$$

thus, sound waves propagate with spherical wavefronts and multiple reflections occur, which cause interference and as the difference between *λ* and *l*_*T*_ increases, the significance of the interference also increases (see Ref. [25], chapter 13). As a result, the magnitude of *σ*_*τ*_ also increases.

**Fig. 5:**
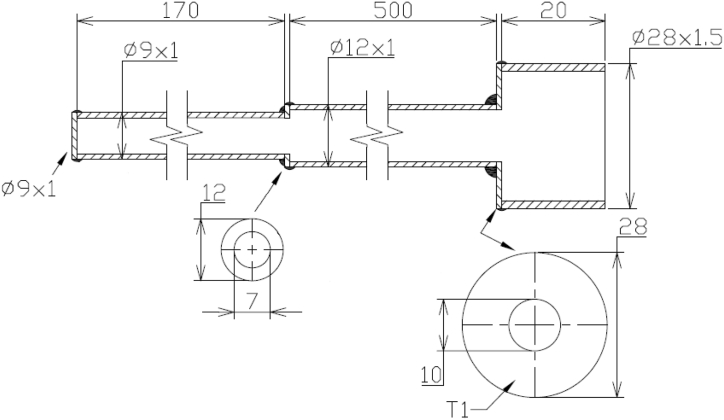
The design of the waveguide that is currently used.

A slightly thinner waveguide replaced the original one to avoid the operation of PAT in the audio-frequency band. Its design is shown in Fig. 5 and theoretically allows operation at frequencies ranging from 590 Hz to 29.63 kHz at −20 °C and from 710 Hz to 35.49 kHz at 90 °C (according to Eq. (5) and Eq. (6)). Finally, the working frequency was set to 25 kHz.

**Fig. 6:**
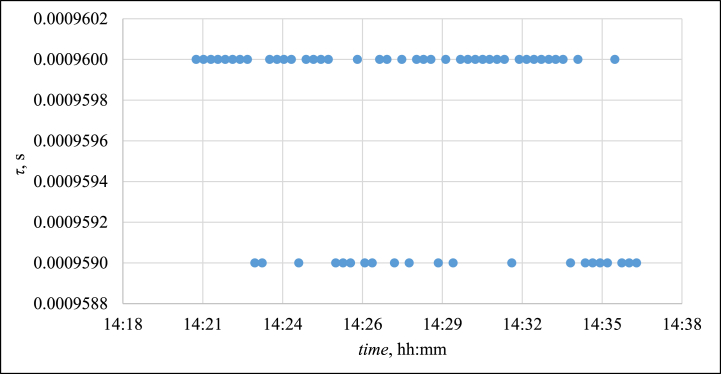
The final time resolution of PAT employing the NI PXI-4461 module, measurement at 49.8 °C.

The NI PXI-4461 module served for the measurement of the microphonic output in Ref. [22]. Even when considering the interpolation of measured data (as recommended in Ref. [12]), the best achievable time resolution of the system was 1 μs (see example in Fig. 6).

**Fig. 7:**
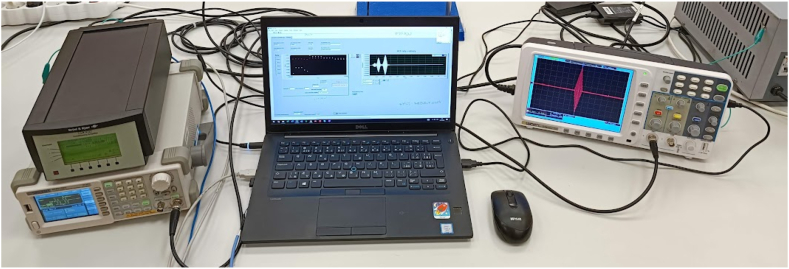
Instrumentation currently used to support the operation of PAT.

The original instrumentation was substituted with the Siglent SDG1025 Waveform Generator and the OWON SDS8302 300 MHz, 2.5 GSa/s digital oscilloscope. Furthermore, the Microtech Gefell MN 921 units were replaced by Bruel & Kjaer 2690-A NEXUS Conditioning Amplifier. The final setup of instrumentation supporting PAT is shown in Fig. 7.

**Fig. 8:**
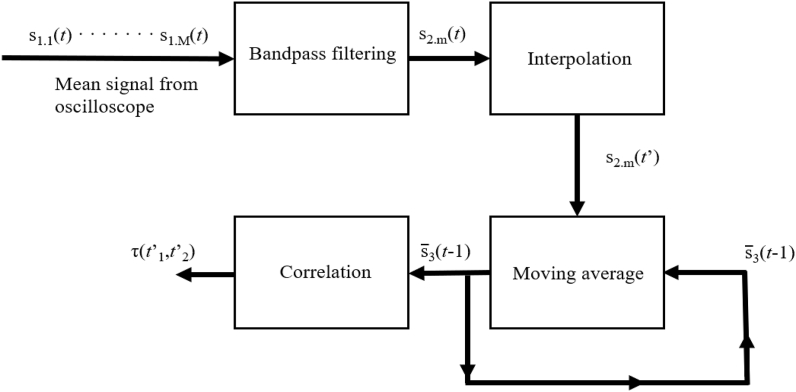
The current PAT measurement process.

The NI PXI-4461 module was measuring the voltage at its input channel continuously. Compared to that, the oscilloscope provides the data vector of limited length. The current measurement procedure is depicted in Fig. 8. While the excitation pulse is repeatedly produced by the generator, the oscilloscope focuses on a 7.6 ms wide area in signal from the conditioning amplifier. Data captured and averaged (by the oscilloscope) represent one period of the acoustic signal radiated by the microphone and reflections (echoes) from the waveguide. Bandpass filtering, interpolation and the average filtering follow. The correlation method of *τ* determination remains in use as well as in Ref. [22].

Parameters of the excitation burst (used in Ref. [22]) were also optimised. The amplitude was raised from 10 V_PP_ to 20 V_PP_, reducing the noise in the signal significantly. The sinusoidal part of the burst was shortened from 0.5 ms to 0.3 ms and the “silent interval” (described in Ref. [22]) was shortened from 24 ms to 6 ms. The resulting time resolution of the system is about 12.5 ns, which corresponds to a resolution in temperature of about 6.35 mK at −20 °C and about 9.1 mK at 90 °C.

Yokogawa DPharp EJX310A pressure gauge with transmitter and Tinsley 5187SA SPRT (s.n. 268711), Tinsley 5187SA SPRT (s.n. 238690) and Fluke 5683 SPRT (s.n. 4219) connected to the WIKA CTR9000 resistance ratio bridge (s.n. 022759/01) with two Tinsley 5685A electrical resistance standards (25 Ω nominal resistance with s.n. 280158 and 100 Ω nominal resistance with s.n. 279875) were used to measure *Δτ* in the temperature range from −80 °C to 220 °C.

**Fig. 9:**
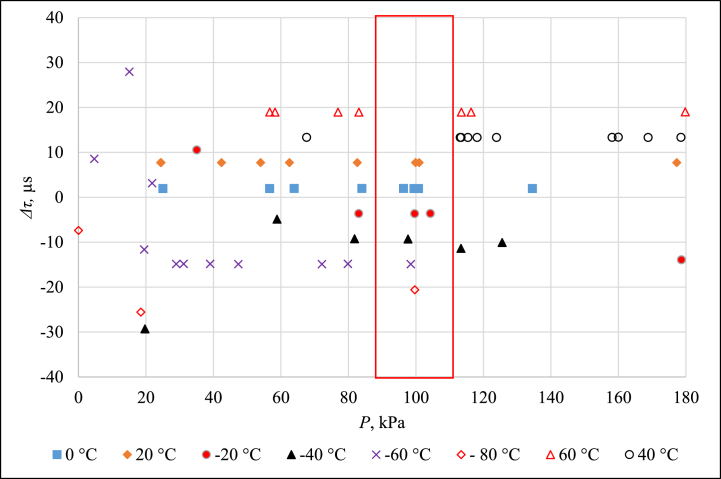
Δτ measurement results from −80 °C to 60 °C.

**Fig. 10:**
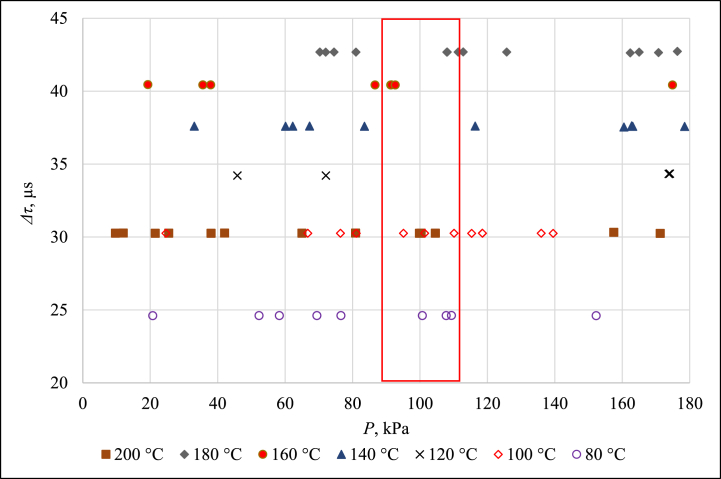
Δτ measurement results from 80 °C to 200 °C.

Three devices served as temperature-controlled environments for this comparison. Fluke 7381 with ethanol filling covered the range from −80 °C to 20 °C, Tamson T.X.V.M.B.70/230 bath with distilled water filling covered the range from 40 °C to 80 °C and Pemit OL250 bath with silicone oil filling covered the range from 80 °C to 220 °C. Since measurements at 220 °C were connected with significantly high standard deviation, the results of *Δτ* measurement that are presented in Fig. 9, Fig. 10 represent temperature range from −80 °C to 200 °C.

**Fig. 11:**
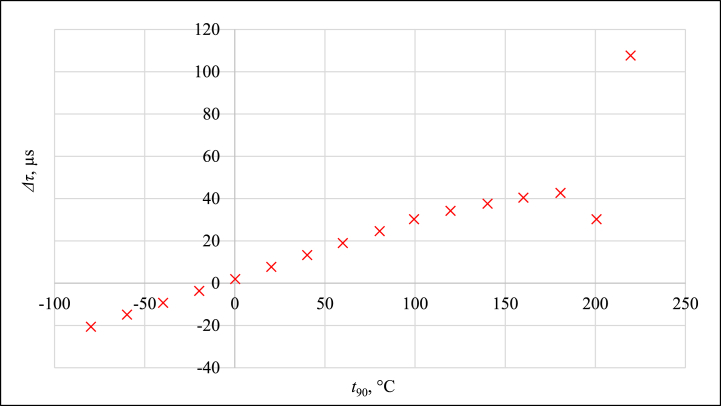
Δτ measurement results for pressure close to 100 kPa.

It is evident that at pressures near the value of 100 kPa, the pressure itself has almost no influence on *Δτ*. Therefore, the measurement results that lie inside the red rectangle in Fig. 9, Fig. 10 are summarized as a characteristic function of the current version of PAT in Fig. 11.

**Fig. 12:**
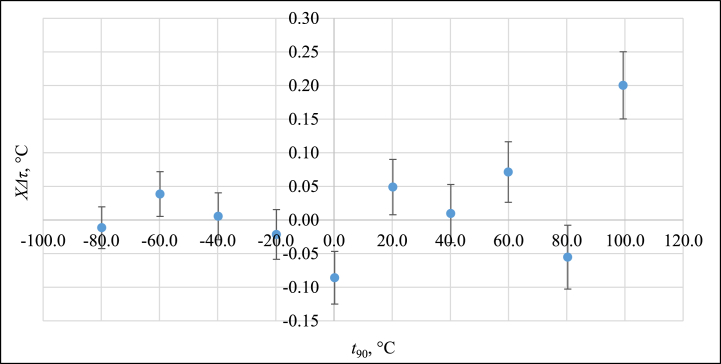
Fitting error with measurement uncertainty, expressed in °C.

These results can be fitted by the polynomial of the second order with *XΔτ* = *Δτ*_measured_ - *Δτ*_fitted_ fitting error ranging from −0.1 °C to 0.1 °C in the temperature range from −80 °C to 120 °C. For this temperature region, the PAT exhibit a second-order dependence of *Δτ* on *t*_*90*_. Fitting error with associated measurement uncertainty (*k* = 2) can be found in Fig. 12, expressed in °C.

It is visible that in the range from −80 °C to 80 °C the PAT follows the IEC 60751 requirements for IPRT tolerance class A with fitting error (combined with measurement uncertainty ranging from 29 mK to 43 mK) not exceeding 0.15 °C. Total measurement uncertainty budgets can be found in Annex A in Table 1, Table 10, Table 2, Table 3, Table 4, Table 5, Table 6, Table 7, Table 8, Table 9

## 3 Conclusion

This academic research marks a significant step in the further development of the practical acoustic thermometer (PAT) at the Czech Metrology Institute, mainly due to its adaptability to argon gas measurements. Through a careful comparison with standard platinum resistance thermometers, the PAT proves to be a promising reference instrument for industrial thermometer calibration, ensuring traceability to the thermodynamic temperature scale. The extended temperature range from −80 °C to 80 °C shows a remarkable reduction in measurement uncertainty, reaching values below 0.15 °C.

However, the challenges become apparent beyond 100 °C, where the microphone approaches the maximum allowable temperature and leads to recognisable differences in the *τ* measurement results. This limitation leads us to consider how we can redesign the microphone to overcome the upper temperature limits. The proposed modifications include changes to the waveguide cover to enable isothermal measurements under elevated pressure and to facilitate the calculation of the speed of sound (*u*_0_). In addition, strategies such as extending the waveguide or installing a special high-temperature microphone are being researched in order to further extend the upper temperature limit.

The novelty of this work lies in contributions to the operational capabilities of the PAT that go beyond conventional limits and extend its utility. These improvements make the PAT a versatile instrument that can cover a wider range of temperatures, increasing its relevance for industrial applications and calibration procedures.

## Data availability statement

The data associated with this study has not been deposited into a publicly available repository. Data will be made available on request.

## Ethics statement

Review and/or approval by an ethics committee was not needed for this study because this study describes experimental device for furnace/bath temperature measurement.

## CRediT authorship contribution statement

**Michal Voldán:** Writing – review & editing, Writing – original draft, Visualization, Validation, Supervision, Software, Resources, Project administration, Methodology, Investigation, Formal analysis, Data curation, Conceptualization. **Libor Husník:** Supervision, Methodology, Conceptualization. **David Mahovský:** Visualization, Investigation, Data curation.

## Declaration of competing interest

The authors declare the following financial interests/personal relationships which may be considered as potential competing interests: Michal Voldan reports financial support was provided by 10.13039/501100004578Ministry of Industry and Trade of the Czech Republic. Michal Voldan reports a relationship with Czech Metrology Institute that includes: employment. This work was funded by Institutional Subsidy for the Long-Term Conceptual Development of a Research Organization granted to the Czech Metrology Institute by the 10.13039/501100004578Ministry of Industry and Trade.

## Figures and Tables

**Table 1:**
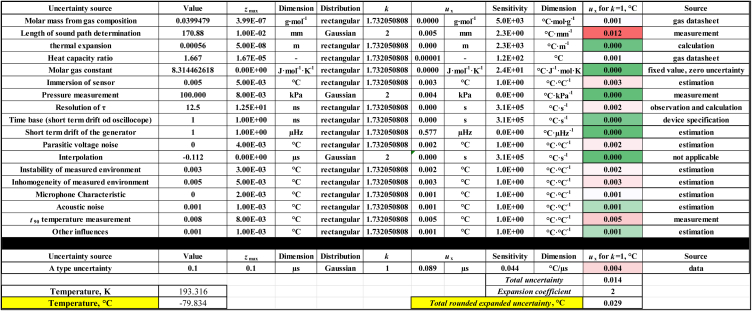
Measurement uncertainty budget for the nominal temperature of −80 °C

**Table 2:**
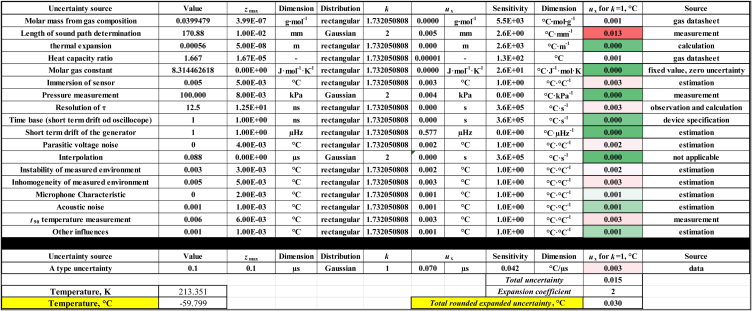
Measurement uncertainty budget for the nominal temperature of −60 °C

**Table 3:**
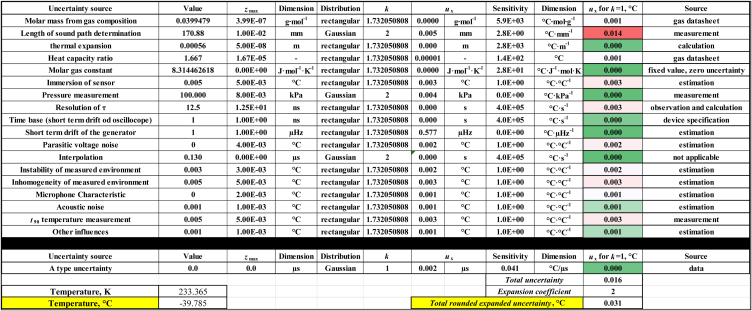
Measurement uncertainty budget for the nominal temperature of −40 °C

**Table 4:**
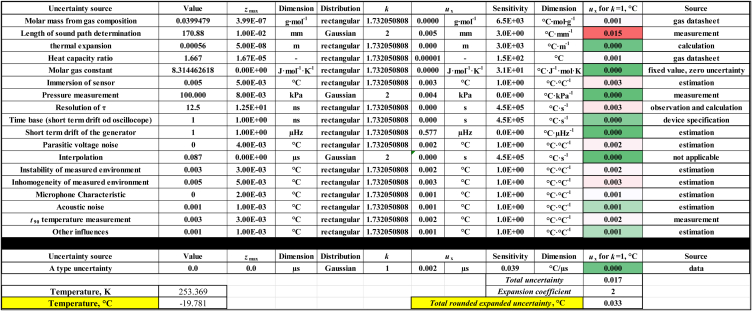
Measurement uncertainty budget for the nominal temperature of −20 °C

**Table 5:**
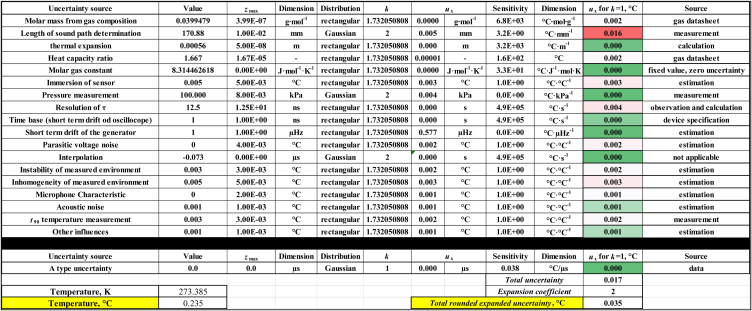
Measurement uncertainty budget for the nominal temperature of 0 °C

**Table 6:**
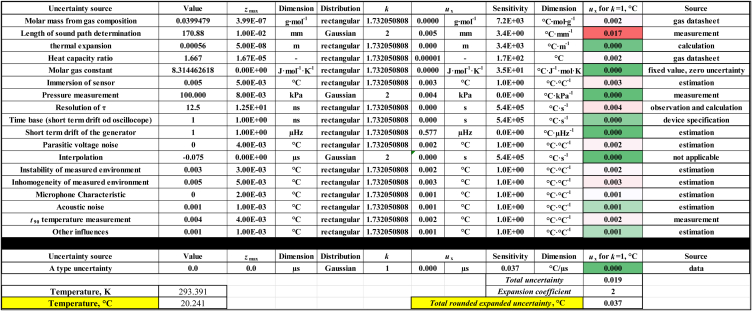
Measurement uncertainty budget for the nominal temperature of 20 °C

**Table 7:**
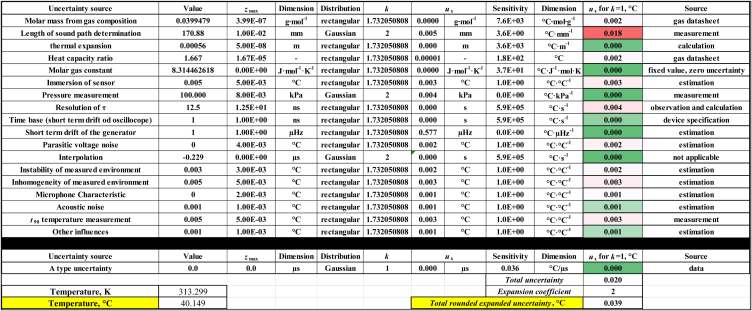
Measurement uncertainty budget for the nominal temperature of 40 °C

**Table 8:**
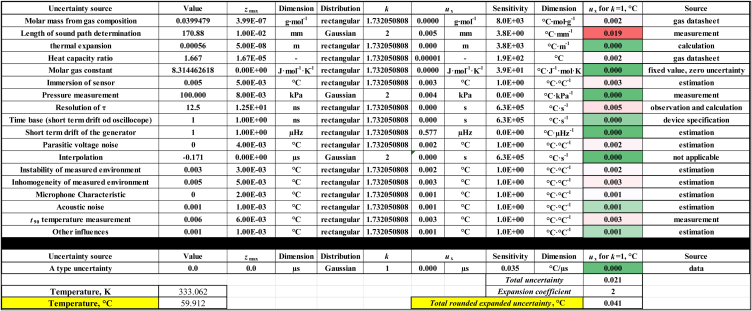
Measurement uncertainty budget for the nominal temperature of 60 °C

**Table 9:**
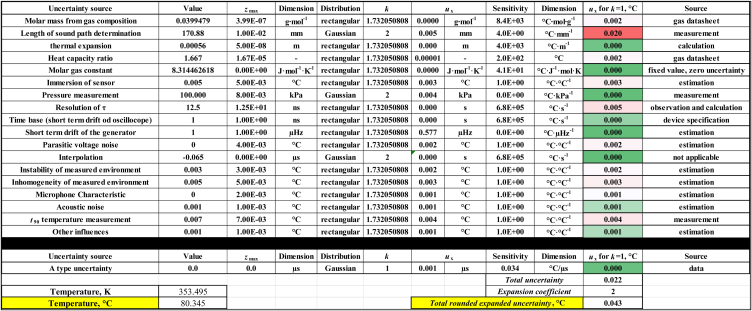
Measurement uncertainty budget for the nominal temperature of 80 °C

**Table 10:**
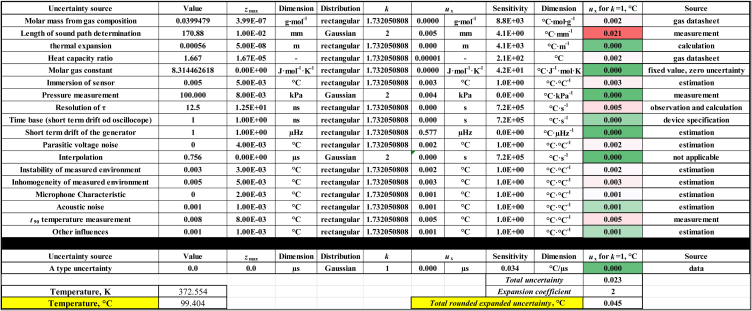
Measurement uncertainty budget for the nominal temperature of 100 °C
